# A multi-faceted community intervention is associated with knowledge and standards of workplace mental health: the Superior Mental Wellness @ Work study

**DOI:** 10.1186/s12889-019-6976-x

**Published:** 2019-05-24

**Authors:** Vicki L. Kristman, Jessica Lowey, Lynda Fraser, Susan Armstrong, Silva Sawula

**Affiliations:** 1 EPID@Work Research Institute, Lakehead University, Thunder Bay, Ontario Canada; 2 Department of Health Sciences, Lakehead University, Thunder Bay, Ontario Canada; 3 Division of Human Sciences, Northern Ontario School of Medicine, Thunder Bay, Ontario Canada; 4 Institute for Work & Health, Toronto, Ontario Canada; 5 Thunder Bay District Health Unit, Thunder Bay, Ontario Canada

**Keywords:** Workplace, Mental health, Community intervention, Mental health literacy, Employee psychological health and safety

## Abstract

**Background:**

Poor psychosocial work environments, such as those with low psychological support and high demands, can be harmful to the mental health of workers. In Canada, the National Standard for Psychological Health and Safety in the Workplace (the Standard) provides a comprehensive framework for organizations to identify hazards that may contribute to the psychological harm of employees. This study examines the association between a multi-faceted community intervention, the Superior Mental Wellness @ Work program designed to increase awareness of mental health and the National Standard, and outcomes assessing increased awareness and response to the Standard. These outcomes included the 1) prioritization of workplace mental health; 2) familiarity with the Standard; and 3) knowledge of mental health.

**Methods:**

A quasi-experimental design was used to assess the associations of interest. Surveys were sent to two random samples of employer representatives pre-and post-intervention. Intervention participants were also compared to non-participants at the post-intervention stage. T-tests and chi-square tests were used to compare differences between pre- and post-intervention outcomes and also between intervention participants and non-participants identified at the post-intervention survey.

**Results:**

The multi-faceted community intervention was associated with increased familiarity of the Standard, and increased knowledge of mental health challenges, mental health promotion, and existing resources at a community-level. When comparing those companies who participated in the intervention versus those who did not, participants were more likely to prioritize mental health in the workplace. Participants reported a greater need for support to address workplace mental health, poorer perceived mental health of employees, and greater stigma than non-participants. However, participants were more likely to be familiar with the Standard, have an action plan to implement the Standard, and be prepared to champion mental health in the workplace. Participants also had greater knowledge of workplace mental health in general compared to non-participants.

**Conclusions:**

The multi-faceted community intervention, the Superior Mental Wellness @ Work project, was associated with increased familiarity of the Standard, and increased knowledge of mental health challenges, mental health promotion, and existing resources at a community-level. Such a multi-faceted intervention has the capacity to improve mental health literacy and awareness of the Standard.

## Background

Common mental disorders are major contributors to the global burden of disease [1]. One in every five people will experience a common mental disorder at some point in their life [2]. Common mental disorders include depression, post-traumatic stress disorder (PTSD), panic disorder, and anxiety [2]. The annual cost of major depressive disorder alone was estimated to be approximately $210.5 billion in 2010 in the US, with 45–47% attributable to direct costs, 5% to suicide-related costs, and 48–50% to workplace costs [3], those being primarily (88%) lost productivity [4].

Common mental disorders have surpassed musculoskeletal disorders as the number one health concern in the workplace. At any one time, 10 to 12 % of the working population is experiencing a mental health disorder [5, 6]. Depression and simple phobia are the most prevalent disorders in the working population and are consistently associated with presenteeism (i.e., lost productivity while at work) over absenteeism [7]. In Canada, the annual productivity impact of mental illness in the workplace was estimated to be over $6.4 billion in 2011, increasing to $16 billion in 2041 [8]. PTSD and panic disorder have the highest rates of mental health service use [8]. Other than simple phobia, all anxiety disorders are associated with impairment in workplace performance [8]. Common mental disorders affect everyone, even if workers are personally not experiencing a mental disorder; they often have a family member, friend or co-worker who is.

Substantial research has shown that jobs with poor psychosocial work environments, those that include workplace factors associated with poor psychological health and safety, can be harmful to the mental health of workers [6, 9–12]. Karasek and Theorell’s demand- control model hypothesizes that high job stress (high job strain and low control over the job environment) will be harmful to health [13]. Using this model, many studies have shown that high job stress is associated with risk for common mental disorders among employees [9–11, 14–16].

In addition to the negative effects of job stress on both employees and employers, there is mounting legal pressure for employers to ensure a psychologically safe workplace. “Changes in labour law, occupational health and safety, employment standards, workers compensation, the contract of employment, tort law, and human rights decisions are all pointing to the need for employers to provide a psychologically safe workplace. In addition, human rights require a duty to accommodate mental disabilities.” [17] Hence, there is a strong need for employers to improve the psychosocial work environment of their workplace.

Common workplace interventions for promoting mental health in the workplace include: cognitive behavioural therapy (CBT)-based interventions; physical activity or relaxation training interventions to reduce stress; and skill- building/training courses to engage employees in their work (job design is a common component) [18]. A recent systematic review synthesized evidence from 11 studies (one service coordination study and 10 multi-domain studies) examining the effectiveness of workplace-based return-to-work (RTW) interventions and work disability management for mental health [19]. The multi-domain studies included components from health-focused interventions, service coordination interventions, or work modification interventions. The findings suggest that: work- focused CBT interventions can help reduce lost time and costs associated with work disability for common mental disorders; and multi-domain interventions for mental health conditions improve work functioning after return-to-work [19]. Hence, integrated intervention approaches to workplace mental health have been recommended to combine the strengths of medicine, public health and psychology, and optimize the prevention and management of mental health problems in the workplace [20].

In Canada, one such integrated intervention is the National Standard for Psychological Health and Safety in the Workplace (the Standard) [21]. The Standard provides a comprehensive framework for organizations to identify hazards that may contribute to the psychological harm of employees. It was developed “in the context of a large body of scientific literature from many relevant areas of workplace health and safety, law, and social science.” [21] The Standard was established to help organizations strive towards the vision of a psychologically healthy and safe workplace that actively works to prevent harm to worker psychological health, including in negligent, reckless or intentional ways, and promotes psychological well-being. It addresses the psychological health and safety aspects within the control, responsibility, or influence of the workplace that can have an impact within, or on, the workforce. This includes the way people interact on a daily basis, working conditions, management practices, and the way decisions are made and communicated.

The Standard outlines 13 identified and measureable workplace factors that have the potential to impact worker mental health, psychological safety, and participation (Table 1) [21]. Implementation of the Standard requires a multi-step process which applies universally, but the actions within the process should be customized to meet the unique needs of each workplace. Currently the guidelines set in the Standard are voluntary and thus workplaces unaware of the Standard will not implement it. Therefore, we developed the Superior Mental Wellness @ Work program, a multi-faceted community intervention designed to increase workplace awareness of mental health and the Standard and to encourage employers to take steps to implement the Standard. We sought to evaluate the impact of the multi-faceted intervention at both the community and program participant level. Therefore, the aim of this study was to determine the association between a multi-faceted community intervention, the Superior Mental Wellness @ Work program, and outcomes assessing increased awareness and response to the Standard. These outcomes included the 1) prioritization of workplace mental health; 2) familiarity with the Standard; and 3) knowledge of mental health. We assessed the intervention on a sample of workplaces from the community by comparing outcome measures pre- and post-intervention and also comparing outcomes to those workplaces who did and did not participate in at least one aspect of the intervention.

**Table 1:** The 13 workplace factors included in Canada’s National Standard for Psychological Health and Safety in the Workplace

Workplace factor	Definition
Organizational culture	The work environment is characterized by trust, honesty and fairness.
Psychological and social support	Co-workers and supervisors are supportive of employees’ psychological and mental health concerns, and respond appropriately as needed. Employees perceive and are aware of organizational support.
Clear leadership and expectations	There is effective leadership and support that helps employees know what they need to do, how their work contributes to the organization and whether there are potential changes.
Civility and respect	Employees are respectful and considerate in their interactions with one another, as well as with customers, clients and the public.
Psychological demands	There is a good fit between employees’ interpersonal and emotional competencies, and the requirements of the position they hold.
Growth and development	Employees receive encouragement and support in the development of their interpersonal, emotional and job skills.
Recognition and reward	There is appropriate acknowledgement and appreciation of employees’ efforts in a fair and timely manner.
Involvement and influence	Employees are included in discussions about how their work is done and how important decisions are made.
Workload management	Tasks and responsibilities can be accomplished successfully within the time available.
Engagement	Employees enjoy and feel connected to their work and feel motivated to do their job well.
Balance	There is recognition of the need for balance between the demands of work, family and personal life.
Psychological protection	Employee psychological safety is ensured.
Protection of physical safety	Management takes appropriate action to protect the physical safety of employees.

## Methods

### Quasi-experimental design

We used a quasi-experimental design to determine the effects of the intervention on mental health attitudes and knowledge of employers in Thunder Bay and the surrounding district. To establish baseline measures, an electronic survey was distributed to a random sample of employers in the Thunder Bay District from September 2016 to November 2016. The Thunder Bay District includes the city of Thunder Bay and the surrounding area from Thunder Bay to Manitouwadge. All employer participants were randomly selected for the study from a list of employers within the District. The list was created by merging two employer lists: 1) from an existing list of employer contacts held at the Thunder Bay District Health Unit, and 2) from a list of employers created through the web-based business data service, Sales Genie [22]. This database includes contact information for employers by geographical area and industrial sector for businesses to use to reach out to prospective clients. It is updated at least every 2 years.

After the baseline data were collected, the multi- faceted community intervention was carried out over a 2-year period. At the end of the intervention period, a follow-up survey was distributed (from April 2018 to July 2018) to another random sample of the same base population to determine if community-level measures had changed by the end of the intervention period. The project was reviewed and approved by the Lakehead University Research Ethics Board (REB Project #151 17–18).

### Characteristics of participants

Study participants were identified from randomly selected employers from Thunder Bay and the surrounding district. Worksites were contacted by either telephone or email depending on the availability and informed of the study. One representative from each worksite was invited to participate. We largely invited employer representatives employed in a human resources or occupational health and safety management role to participate. If worksites did not have these specific roles within their workplace, we invited individual representatives who would have knowledge about employee mental health within their workplace (e.g., general/floor manager, supervisor, etc.), to participate. Worksites interested in participating provided the research team with the contact name and email of a workplace representative. All representatives were emailed a study invitation as well as a link to the online informed consent and survey. Interested participants completed the electronic informed consent and survey. Study data were collected and managed using REDCap electronic data capture tools hosted at Lakehead University [23].

### Baseline and follow-up surveys

Baseline and follow-up surveys included similar questions addressing: state of current workplace mental health and stigma in the workplace; familiarity with the Standard; and measures assessing general knowledge levels related to mental health in the workplace. Six questions addressed the current state of mental health in the workplace. These questions were derived from the workplace mental health in Canada survey led by the Canadian Mental Health Association and the Workforce Mental Health Collaborative [24]. Four questions assessed familiarity and level of commitment to implementing the Standard in the workplace. Eight questions addressed level of knowledge related to mental health and were measured on a five-point scale from “Not at all knowledgeable” to “Extremely knowledgeable” (Table 1). The latter 12 questions were derived by the research team. These questions were pilot tested for face validity, but were not formally assessed in a validation study.

The baseline survey was distributed in the fall of 2016. The first training session had started earlier that spring. Ten (31%) of those in the first training session also completed a baseline survey.

### Multi-faceted intervention

The multi-faceted intervention included three separate components that were offered at various times over the 2-year intervention period: 1) a six-session *Standard to Action* training program designed to help employers implement the Standard in their workplaces; 2) education and training sessions involving various experts to discuss topics related to workplace mental health; and 3) a social marketing campaign including a photovoice exhibit that was developed from photos and captions submitted by community members related to a) how people really feel at work and b) how people take care of their mental health at work.

**Fig. 1:**
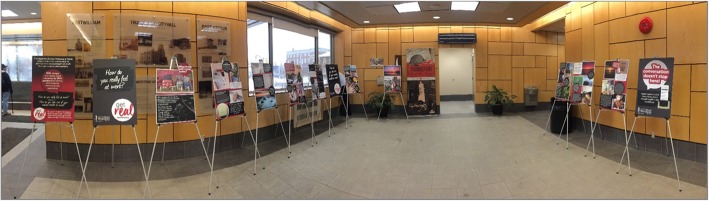
The *Get Real* photovoice exhibit (photo courtesy of Krystina Hunter, City of Thunder Bay)

1. The “Standard to Action” training program was offered three times over the intervention period: March to October 2016; March to October 2017; and September 2017 to February 2018. Offerings one and three were held in Thunder Bay, Ontario, while the second offering was held in Nipigon, Ontario (approximately 100 km east of Thunder Bay). Each offering was comprised of six sessions, occurring approximately once per month. The goal of the training program was to increase the number of workplace environments that maintain positive mental health for employees (i.e., create a workplace environment where workers can flourish and maintain low levels of mental health disorders). The program was based on a training program developed by Workplace Safety and Prevention Services and had six objectives: a) increase understanding of the Standard and its purpose in the workplace; b) development (and implementation) of customized action plans for implementing the Standard; c) increase the number of workplaces with a mental health policy/commitment; d) reduce mental health stigma in workplaces; e) increase networking opportunities to share ideas and challenges; and f) prepare participants to serve as ambassadors of the Standard within their organizations.
2. The education and training sessions included a speaker series, Mental Health First Aid courses and Mental Health Works sessions. The speaker series included 10 speakers over 11 events. Topics included work-life balance, psychologically safe workplace conversations, how to build a positive workplace culture, stress and resiliency, reducing mental health stigma, mental health awareness, accommodations for mental health in the workplace, and how managers should respond. Four sessions of Mental Health First Aid were offered through the Mental Health Commission of Canada’s courses. Three sessions of Mental Health Works were offered in Thunder Bay. Mental Health Works is offered by the Canadian Mental Health Association. The overall aims of these training programs are to build mental health awareness, strengthen ability to respond to challenging situations, and foster healthier, safer workplace environments [25].
3. The social marketing campaign and photovoice exhibit aimed to reduce the stigma associated with poor mental health and to spark meaningful conversations about mental health in the workplace. The exhibit was developed in June of 2017 by asking the communities within the Thunder Bay District to answer two questions using an anonymous photo and a caption describing the meaning behind the photo. The two questions were: a) “How do you really feel at work?”; and b) “How do you take care of your mental health at work?” The campaign and exhibit (Fig. 1) were developed from the submitted photos. The exhibit was made available for workplaces to sign out and display within their organization for a week at a time.

All components of the intervention were widely advertised to employers across Thunder Bay and the surrounding district through the Thunder Bay District Health Unit’s website, social media channels, and direct emails to workplace wellness contacts. Interested employers registered participants to attend the training sessions, education events, and to display the photovoice exhibit in their workplace. The photovoice exhibit was on display at local health and safety conferences in Thunder Bay, Thunder Bay City Hall, Thunder Bay District Health Unit, and in 17 additional worksites between October 2017 and May 2018. There was no limit on the number of events employers and employees could participate in.

### Statistical analyses

We examined the data for missing responses, erroneous entries and illogical responses by examining frequency distributions to detect missing and outlying information. Wherever possible, we replaced erroneous information with correct information. If we could not discern the correct information, we categorized the data point as missing. We ran basic descriptive statistics on participant characteristics to understand who participated in the pre- and post-intervention surveys. It was also determined, among those who completed the post- intervention survey, how many participated in some aspect of the multi-faceted intervention.

To assess the impact of the intervention on overall community-level outcomes (prioritizing workplace mental health, familiarity with the Standard, and knowledge of mental health), we used t-tests to compare mean differences for continuous variables and chi-square tests to assess proportional differences in categorical variables between pre- and post-intervention outcomes.

To further determine the effectiveness of the intervention itself, we compared those who participated in some aspect of the intervention to those who did not among those who completed the post-intervention survey. These comparisons were also conducted with t-tests and chi-square tests. All analyses were conducted using SPSS version 24 [26].

## Results

### Participating employers

We invited a total of 319 randomly-selected companies to participate in the pre-intervention survey and 350 to participate in the post-intervention survey. We received 89 and 61 completed surveys for the pre- and post- intervention surveys, for response rates of 28 and 17.4%, respectively. Table 2 shows the characteristics of the randomly selected companies that completed the surveys. The post-intervention surveys are stratified by those who were active participants in the intervention (meaning they attended any of the training programs or speaker events, or promoted the photovoice exhibit in their workplace) or not. Companies who completed surveys were most likely to be from white collar workforces (health care, education, or professional sectors). However, the breakdown of participants by company size was more evenly distributed with the largest proportion in the 100 to 250 employee category.

**Table 2:** Distribution of participant characteristics in the pre- and post-intervention samples

Characteristic	Pre-intervention (*N* = 89)	Post-intervention (*N* = 61)	
		Intervention participants (*N* = 37)	Intervention non-participants (*N* = 24)
Industrial sector			
1. Mining, etc.; Utilities; Construction	8 (9.0)	2 (5.4)	3 (12.5)
2. Manufacturing	2 (2.2)	–	1 (4.2)
3. Trade (Wholesale, Retail); Transportation	5 (5.6)	2 (5.4)	2 (8.3)
4. Information and cultural industries; Finance/insurance; Real Estate, etc.	2 (2.2)	1 (2.7)	–
5. Professional, scientific, etc.	10 (11.2)	6 (16.2)	6 (25.0)
6. Educational	14 (15.7)	6 (16.2)	6 (25.0)
7. Arts, entertainment, etc.	6 (6.7)	–	2 (8.3)
8. Health care and social assistance	18 (20.2)	12 (20.2)	1 (4.2)
9. Other services (except public administration)^a^	14 (15.7)	5 (13.5)	1 (4.2
10. Public administration	5 (5.6)	1 (2.7)	2 (8.3)
11. Missing	5 (5.6)	2 (5.4)	–
Company Size			
Under 10	12 (13.5)	1 (2.7)	7 (29.2)
10 to 24	16 (18.0)	5 (13.5)	3 (12.5)
25 to 49	13 (14.6)	5 (13.5)	6 (25.0)
50 to 99	12 (13.5)	5 (13.5)	2 (8.3)
100 to 250	23 (25.8)	16 (43.2)	4 (16.7)
Over 250	7 (7.9)	3 (8.1)	2 (8.3)
Missing	6 (6.7)	2 (5.4)	–
Participant Gender			
Male	59 (66.3)	31 (83.8)	13 (54.2)
Female	24 (27.0)	4 (10.8)	11 (45.8)
Missing	6 (6.7)	2 (5.4)	–
Participant Age Group			
18 to 30	9 (10.1)	2 (5.4)	2 (8.3)
31 to 40	35 (13.5)	5 (13.5)	5 (20.8)
41 to 50	6 (29.2)	12 (32.4)	5 (20.8)
51 to 60	6 (38.2)	15 (49.5)	10 (41.7)
Over 60	5 (3.4)	–	2 (8.3)
Missing	5 (5.6)	3 (8.1)	–
Participant Position			
Upper Management	37 (41.6)	10 (27.0)	13 (54.2)
Middle Management	35 (39.3)	18 (48.6)	7 (29.2)
Front Line Worker	6 (6.7)	4 (10.8)	2 (8.3)
Other	6 (6.7)	3 (8.1)	2 (8.3)
Missing	5 (5.6)	2 (5.4)	–
Participant Employment Length			
Less than a year	3 (3.4)	1 (2.7)	1 (4.2)
1 to less than 2 years	3 (3.4)	1 (2.7)	3 (12.5)
2 to less than 5 years	14 (15.7)	6 (16.2)	3 (12.5)
5 to 10 years	11 (12.4)	8 (21.6)	1 (4.2)
Over 10 years	51 (57.3)	18 (48.6)	16 (66.7)
Missing	7 (7.9)	3 (8.1)	

^a^Other services (except public administration) is defined by the NAICS 2012 and comprises of only establishments, not classified to any other sector including repair/maintenance, personal and laundry services, religious organizations, private households, etc.

Thirty-seven employers reported actively participating in some aspect of the intervention. Of these, 81% participated in the training program, 70% attended a speaker event, and 40% viewed or hosted the photovoice exhibit. Approximately 38% participated in at least two of the three aspects of the intervention, and 27% participated in all three.

### Community level pre- and post-intervention comparisons

#### Prioritizing workplace mental health

At a community level, the multi-faceted intervention was not associated with prioritizing workplace mental health. Although the differences observed between the pre- and post-intervention scores suggest that employers prioritized mental health in the workplace, and mental health supports are needed, these differences were not statistically significant (Table 3). The direction of the differences also suggested organizations reported less effort to address workplace mental health, less perceived employee mental health, and more perceived stigma in their workplaces after the intervention; however, these differences were also not statistically significant (Table 3).

**Table 3:** Community level pre- and post-intervention scores assessing survey domains

Survey Item	Measurement scale	Pre-intervention mean (SD) *N* = 89	Post-intervention mean (SD) *N* = 61	Mean difference (95% CI)
Prioritizing workplace mental health domain				
1) At this time, how much of a priority is employee mental health for your organization?	0 (lowest priority) – 5 (top priority)	3.08 (1.18)	3.31 (1.03)	0.23
				(− 0.13, 0.59)
2) Is employee mental health an issue that your organization is looking for support to address?	0 (No); 1 (Yes)	0.55 (0.50)	0.72 (0.45)	0.17^a^
				(−0.003, 0.43)
3) At this time, how much support is your organization looking for to address employee mental health?	0 (No support) – 3 (a lot of support)	1.84 (1.34)	2.03 (1.21)	0.21
				(−0.23, 0.61)
4) At this time, how well do you think your organization is doing in its efforts to address workplace mental health?	0 (Not well) – 3 (very well)	1.36 (0.91)	1.36 (0.91)	−0.01
				(−0.31, 0.30)
5) In general, how would you rate employee mental health in your workplace environment?	0 (poor) – 4 (excellent)	1.91 (0.97)	1.89 (0.88)	−0.02
				(−0.33, 0.28)
6) In general, how would you rate the amount of mental health stigma in your workplace?	0 (high) – 3 (no stigma present)	1.84 (0.74)	1.71 (0.64)	−0.13
				(−0.37, 0.11)
The Psychological Health and Safety Standard domain				
1) How familiar are you with the National Standard for Psychological Health and Safety in the Workplace?	0 (not at all) – 4 (extremely familiar)	1.21 (1.36)	1.88 (1.49)	0.67
				(0.20, 1.14)
2) Has your workplace developed an action plan for implementing the Standard?	0 (No) – 2 (Yes, a plan is developed)	0.52 (0.73)	0.57 (0.83)	0.05
				(−0.24, 0.34)
3) Does your workplace have a mental health policy or commitment in place?	0 (No) – 3 (Yes, one is in place)	1.03 (1.29)	1.40 (1.31)	0.37
				(−0.09, 0.84)
4) At this time, how prepared are you to champion mental health in your workplace?	0 (Not prepared at all) – 3 (very prepared)	1.55 (1.01)	1.62 (0.99)	0.09
				(−0.25, 0.42)
Mental health knowledge domain				
Knowledge relating to:				
1) Mental health in general	0 (not at all) – 4 (extremely knowledgeable)	2.13 (0.83)	2.39 (0.74)	0.26
				(−0.01, 0.51)
2) Mental health challenges in the workplace		2.02 (0.88)	2.36 (0.76)	0.33
				(0.06, 0.60)
3) Mental health stigma and its impact		2.08 (0.99)	2.34 (0.74)	0.26
				(−0.02, 0.55)
4) The legal and legislative perspectives around mental health in the workplace		1.43 (1.08)	1.58 (1.00)	0.15
				(−0.19, 0.49)
5) Accommodation of workers with mental illness		1.78 (1.06)	1.92 (0.93)	0.13
				(−0.20, 0.46)
6) Mental health promotion strategies		1.43 (1.07)	1.90 (0.94)	0.47
				(0.14, 0.80)
7) Existing resources to support mental health at work		1.63 (1.02)	1.97 (0.86)	0.33
				(0.02, 0.64)
8) How to build a business case to gain management support for mental health		1.19 (1.06)	1.52 (1.08)	0.33
				(−0.03, 0.69)

^a^Chi-square used to compare proportions

#### Familiarity with the National Standard for Psychological Health and Safety in the Workplace (the Standard)

The multi-faceted intervention was associated with familiarity of the Standard (Table 3). However, workplaces were no more likely to report developing an action plan for implementing the Standard, having a mental health policy or commitment in place, nor being prepared to champion mental health in the workplace after the intervention than they were prior to the intervention.

#### Knowledge of mental health

The multi-faceted intervention was associated with three areas of increased knowledge: mental health challenges in the workplace, mental health promotion strategies, and existing resources to support mental health at work. Although knowledge scores in all areas improved from pre-intervention to post-intervention, in only those three areas were the differences statistically significant (Table 3).

### Intervention participant and non-participant comparisons

#### Prioritizing workplace mental health

When comparing those who participated in the multi- faceted intervention to those who did not, those who participated in the program were more likely to report: mental health as an organizational priority, a need for support to address workplace mental health, poorer perceived employee mental health, and higher amounts of negative mental health stigma (Table 4). Intervention participants indicated a higher score in organizational effort than non-participants, but that difference was not statistically significant.

**Table 4:** Post-intervention survey results for program participants and non-participants

Survey Item	Measurement scale	Intervention participants mean (SD) *N* = 37	Non-participants mean (SD) *N* = 24	Mean difference (95% CI)
Prioritizing workplace mental health domain				
1) Mental health priority	0 (lowest priority) – 5 (top priority)	3.58 (0.77)	2.92 (1.22)	0.66
				(0.10, 1.22)
2) Mental health support needed	0 (No); 1 (Yes)	0.87 (0.34)	0.47 (0.51)	0.40^a^
				(0.13, 0.67)
3) Level of support needed	0 (No support) – 3 (a lot of support)	2.11 (0.82)	1.92 (1.63)	0.19
				(−0.53, 0.91)
4) Organizational efforts	0 (Not well) – 3 (very well)	1.44 (0.91)	1.22 (0.90)	0.23
				(−0.26, 0.71)
5) Perceived employee mental health	0 (poor) – 4 (excellent)	1.78 (0.83)	2.04 (0.94)	−0.26
				(−0.73, 0.21)
6) Perceived mental health stigma	0 (high) – 3 (no stigma present)	1.52 (0.57)	2.00 (0.63)	−0.48
				(−0.83, − 0.14)
The Psychological Health and Safety Standard domain				
1) Familiarity with the Standard	0 (not at all) – 4 (extremely familiar)	2.60 (1.31)	0.88 (1.09)	1.72
				(1.10, 2.34)
2) Standard action plan development	0 (No) – 2 (Yes, a plan is developed)	0.83 (0.91)	0.19 (0.51)	0.64
				(0.24, 1.05)
3) Mental health policy in place	0 (No) – 3 (Yes, one is in place)	1.55 (1.26)	1.16 (0.32)	0.39
				(−0.40, 1.18)
4) Mental health ambassador readiness	0 (Not prepared at all) – 3 (very prepared)	1.97 (0.81)	1.13 (0.21)	0.85
				(0.34, 1.35)
Mental health knowledge domain				
Please indicate your current level of knowledge relating to:				
1) Mental health in general	0 (not at all) – 4 (extremely knowledgeable)	2.57 (0.66)	2.13 (0.80)	0.45
				(0.05, 0.84)
2) Mental health challenges		2.54 (0.56)	2.08 (0.93)	0.46
				(0.03, 0.89)
3) Mental health stigma and its impact		2.54 (0.61)	2.04 (0.83)	0.50
				(0.09, 0.91)
4) The legal perspectives		1.86 (0.88)	1.17 (1.05)	0.69
				(0.17, 1.22)
5) Accommodation for mental illness		2.09 (0.82)	1.67 (1.05)	0.42
				(−0.10, 0.93)
6) Mental health promotion strategies		2.29 (0.75)	1.33 (0.92)	0.95
				(0.50, 1.41)
7) Existing resources		2.15 (0.61)	1.71 (1.08)	0.44
				(−0.06, 0.94)
8) Building a business case		1.88 (1.15)	1.00 (0.72)	0.88
				(0.39, 1.38)

^a^Chi-square used to compare proportions

#### Familiarity with the National Standard for Psychological Health and Safety in the Workplace (the Standard)

Intervention participants were much more likely to report familiarity with the Standard (Table 4). They were also more likely to have an action plan in place for implementing the Standard and were more prepared to champion mental health in the workplace. However, intervention participants were no more likely to have a mental health policy in place than non-participants.

#### Knowledge of mental health

Intervention participants scored significantly higher on all mental health knowledge questions except for knowledge of accommodations and knowledge of existing resources (Table 4). Intervention participants reported greater knowledge than non-participants on mental health in general, mental health challenges, stigma and its impact, legal perspectives, mental health promotion strategies, and knowledge around how to build a business case to gain management support for mental health promotion in the workplace.

## Discussion

This study found that a multi-faceted community intervention was associated with increased familiarity of the Standard and increased knowledge, including knowledge of mental health challenges, knowledge of mental health promotion, and knowledge of existing resources. When comparing those companies who participated in the intervention versus those who did not, participants were more likely to prioritize mental health in the workplace. Participants reported a greater need for support to address workplace mental health, poorer perceived mental health of employees, and greater stigma than non- participants. However, participants were more likely to be familiar with the Standard, have an action plan to implement the Standard, and be prepared to champion mental health in the workplace. Participants also had a greater knowledge of workplace mental health in general compared to non-participants.

We could not find any other study that examined the infiltration of intervention effects into a community of workplaces. Our study is novel in that we assessed a random sample of workplaces in the community at both pre- and post-intervention to determine how the intervention diffused into the community of workplaces. We found diffusion of information related to familiarity with the Standard and increased knowledge in domains related to mental health.

The only other multi-faceted community intervention we are aware of was conducted in an Australian Macedonian community with the intention of reducing stigma and improving mental health literacy [27]. Although not specific to workplace mental health, this study included presentations of research findings on the comprehension of mental illness and attitudes and stigma levels given in two community and three workplace education sessions [27]. Similar to our findings, they found participants reported increased awareness of stigma, although they did not have a control group to compare these participants to [27].

Other specific programs, such as Mental Health First Aid training, have been shown to improve workplace participants’ mental health literacy [28–30]. However, these studies focus specifically on employees of specific governmental departments [28, 29], and hospital employees [30]. Although many have received Mental Health First Aid training with very positive results on mental health literacy [31], few studies have focused specifically on occupational groups beyond teachers [32], pharmacy students [33], rural support and community workers [34], and public sector managers [35]. Our participant pool was randomly selected from lists of employers in the study population and could include any interested workplace representative from any industrial sector. Our participants included mostly upper and middle management from white collar workforces (health care, education, or professional sectors). Management participants are not surprising as it would likely be someone from management who would be selected to complete a survey on behalf of the organization. White collar workforces with higher education levels may be more mental health literate [36], and therefore, more likely to participate in a survey request on mental health in the workplace. To corroborate this, we found that intervention participants were associated with higher prioritization of mental health in the workplace. We also found that intervention participants were more likely to be familiar with the Standard. It may have been that those with more familiarity of the Standard were more likely to be interested in aspects of the multi-faceted intervention and hence, participated in it. Finally, participants also had a greater knowledge of workplace mental health, but this could also be explained by the levels of knowledge of those who participated in the intervention rather than the intervention itself increasing their education.

### Limitations

The greatest limitation to the interpretation of these findings is that of selection bias. Given the low response rates, it is likely that those with a greater interest in workplace mental health would be more likely to participate in such an intervention. However, results where the non- participants post-intervention score is greater than the score from the random sample at pre-intervention would suggest that the intervention may have had an overall influence on the outcomes. These outcomes include the level of support needed, perceived employee mental health and mental health stigma, knowledge of mental health in general, knowledge of mental health challenges, and knowledge of existing resources. Although these differences are small and non-significant they could be considered intervention effects accounting for selection bias.

Small sample sizes precluded the use of more advanced statistical analyses, such as multi-level modelling. Therefore, we were unable to control for extraneous factors such as company and individual participant factors.

A small number of participants who completed the baseline survey had already attended some of the training sessions. This would tend to make these participants respond more similar to the post-intervention survey respondents, meaning these results are conservative.

Finally, the random selection of participants at pre- and post-intervention prohibits pairwise comparisons and thus any determination of causation by the multi- faceted intervention. Future studies should incorporate the randomization of companies to participate in the multi-faceted intervention. Although randomizing participation may be particularly challenging for some sectors of employers, this information would be invaluable for a process evaluation where it could be determined who is able to access the intervention.

## Conclusion

We found that a multi-faceted community intervention, the Superior Mental Wellness @ Work project, was associated with increased familiarity of the Standard, and increased knowledge of mental health challenges, knowledge of mental health promotion, and knowledge of existing resources at a community-level. Such a multi- faceted intervention has the capacity to improve mental health literacy and awareness of the Standard. Further research is needed to determine the causal nature of the associations found and to determine if there are particular sectors of workplaces where the multi-faceted intervention may be more effective.

## Abbreviations

CBT: Cognitive Behavioural Therapy
PTSD: Post-traumatic Stress Disorder
RTW: Return to Work
The Standard: The National Standard for Psychological Health and Safety in the Workplace
